# Deep learning for predicting 16S rRNA gene copy number

**DOI:** 10.1038/s41598-024-64658-5

**Published:** 2024-06-20

**Authors:** Jiazheng Miao, Tianlai Chen, Mustafa Misir, Yajuan Lin

**Affiliations:** 1 Division of Applied and Natural Sciences, Duke Kunshan University, Suzhou, China; 2 Department of Life Sciences, Texas A&M University-Corpus Christi, Corpus Christi, USA; 3 Department of Biomedical Informatics, Harvard Medical School, Boston, USA; 4 Department of Biomedical Engineering, Duke University, Durham, USA

**Keywords:** Computational biology and bioinformatics, Microbiology

## Abstract

Culture-independent 16S rRNA gene metabarcoding is a commonly used method for microbiome profiling. To achieve more quantitative cell fraction estimates, it is important to account for the 16S rRNA gene copy number (hereafter 16S GCN) of different community members. Currently, there are several bioinformatic tools available to estimate the 16S GCN values, either based on taxonomy assignment or phylogeny. Here we present a novel approach ANNA16, Artificial Neural Network Approximator for 16S rRNA gene copy number, a deep learning-based method that estimates the 16S GCN values directly from the 16S gene sequence strings. Based on 27,579 16S rRNA gene sequences and gene copy number data from the rrnDB database, we show that ANNA16 outperforms the commonly used 16S GCN prediction algorithms. Interestingly, Shapley Additive exPlanations (SHAP) shows that ANNA16 can identify unexpected informative positions in 16S rRNA gene sequences without any prior phylogenetic knowledge, which suggests potential applications beyond 16S GCN prediction.

## Introduction

Culture-independent 16S rRNA metabarcoding is a molecular biology technique that applies DNA sequencing technology to the prokaryotic 16S rRNA gene to identify and quantify the microbial communities present in a sample^1^. Over the last two decades, this technique has emerged as a high-throughput, prevalent method for inferring microbial community structure^2–4^. A major limitation, however, lies in its estimation based on 16S rRNA gene read counts rather than the true cell counts. With prokaryotes having variable numbers of 16S rRNA gene copies per cell (ranging from 1 to 21 copies/genome)^5^, the resultant relative 16S rRNA gene abundance does not accurately reflect the actual microbial community composition^6,7^. To address this issue and depict a more accurate microbiome profile, the proportion of 16S rRNA gene read counts should ideally be adjusted by the inverse of the 16S rRNA gene copy number (hereafter 16S GCN). This would provide a corrected microbial composition that represents the actual proportion of cells of each lineage in the community^6^.

To measure 16S GCN values, researchers typically rely on experimental methods such as whole genome sequencing^5^ or competitive PCR^8^. However, these methods can be expensive and/or culture-dependent, and thus experimental estimation of 16S GCN are only available for a limited number of species. To overcome this limitation, several bioinformatics tools have been recently developed to predict the 16S GCN of yet-to-be-measured species using data from those already measured. While there are some deviations^9^, a prevailing hypothesis is that the 16S GCN correlates with the phylogenetic proximity of species^6,10^. This suggests minimal 16S GCN variations within closely related lineages. Hence, 16S GCN can be inferred from taxonomy or phylogeny^6,10,11^. For instance, rrnDB^5^, a highly cited tool, estimates 16S GCN based on taxonomy, deducing the mean 16S GCN of a particular taxon from the averages of its sub-taxa. Another example, PICRUSt2^12^, employs a phylogenetic tree of the 16S rRNA genes and estimates the 16S GCN of the unmeasured species from its close measured relatives.

Deep learning, as a popular machine learning strategy, has been recently applied to model complex patterns from biological sequencing data^13,14^. In this study, we explore deep learning as a novel approach to predict 16S GCN directly from DNA sequences. Deep learning is a branch of machine learning that employs layers of Artificial Neural Networks (ANN) to learn complex data^15^. The concept of ANN is inspired by human cognitive processes, where information travels and is processed through synaptic interactions between neurons^16^. An ANN is a computational model that is constituted by multiple interconnected processing elements—artificial neurons^16^. Neurons are utilized in groups as the layers of a network. Each artificial neuron receives and processes input with a non-linear function, known as the activation function^17^. Collectively, one ANN model learns to solve a specific task by capturing non-linear trends and relationships from the presented data. Impressively, ANNs can disclose relationships and mechanisms even eluding scientific understanding^18^. Various deep learning architectures, like the Convolutional Neural Network, and Recurrent Neural Network, have been applied to microbiome research, including metagenomic-based taxonomy classification^19^, 16S rRNA classification^20^, host phenotype prediction^21^, disease prediction^22^, and microbial community prediction^23^. Furthermore, deep learning algorithms have been developed to conduct data mining for antibiotic resistance genes^24^, antimicrobial peptides^25^, and microbial sources^26^.

This study investigates the potential of using deep learning to estimate 16S GCN directly from 16S rRNA gene sequences, circumventing informative loss in taxonomy classification and phylogeny. In other words, this study treats the task of 16S rRNA gene copy number correction as a deep-learning regression problem, and aims to develop a deep learning model which takes a 16S rRNA gene sequence as the input, learns the evolutionary information from the sequence, and predicts the copy number of the 16S rRNA gene in the genome as the output.

Out of the various deep learning models examined, an ensemble model of the Multi-layer Perceptron (MLP) and Support Vector Machine (SVM) model was selected. This model was trained on the 16S full-length DNA sequences, and its performance was compared against the traditional taxonomy and phylogeny-based algorithms. Given that microbiome metabarcoding often targets one to three adjacent hypervariable regions within the 16S rRNA^27^, models were also trained and tested on these commonly amplified subregions. Finally, ANNA16, a deep learning-based bioinformatic tool for 16S GCN correction was developed. Its performance was evaluated against current 16S GCN correction tools. Furthermore, the study ventured into interpreting DNA sequence information through the lens of ANNA16, employing Shapley Additive exPlanations (SHAP), a game theory-based model explanation method^28^.

## Materials and methods

### Data source and preprocessing

16S rRNA gene sequences and the 16S GCN data used to train deep learning models in this study were retrieved from rrnDB, version 5.7^5^. The dataset includes a total of 20,277 16S gene sequences with unique accession numbers, containing both ( +) strands and (-) strands.

Firstly, the orientation for all sequences in this dataset was identified using the BLASTN algorithm (BLAST + , version 2.11.0)^29^. The first 50 bp of the *E. coli* 16S rRNA gene sequence ( +) strand (accession number: GCF_002953035.1), a conserved region on 16S rRNA^30^, was used as a template. Sequences with High-Scoring Segment Pairs (HSPs) that had sstart (subject start) > send (subject end) and evalue < 0.01 were identified as ( +) strands, and those with sstart < send and evalue < 0.01 were identified as (-) strands. 389 sequences that did not meet these criteria were labeled as unrecognizable and excluded from the dataset. The reverse complement of these (-) strand sequences was then added to the ( +) strand sub-dataset.

Next, to have uniform starting and ending positions, all 16S rRNA gene sequences in the database were further trimmed by HyperEx^31^, using the universal full-length 16S primer 27F and primer 1492R (Table S1)^27,32^. After that, 19,520 sequences remained in this full-length dataset, involving 1331 genera, with an average sequence length of 1498.72 nucleotides and a standard deviation of 21.41 nucleotides. 16S GCN ranges from 1 to 21 copies/genome (Mean = 5.26, SD = 2.74). In addition, seven commonly targeted subregions by metabarcoding, i.e., V1–V2, V1–V3, V3–V4, V4, V4–V5, V6–V8, and V7–V9^27^, were also extracted by HyperEx (primers listed in Table S1) to generate subregion datasets. The full-length and subregional datasets were used as the training datasets.

By conducting a five-fold cross-validation at each region, the rrnDB 5.7 dataset was first split into training and test sets. This was done to evaluate the performance of various deep learning models, including a MLP-based Stacked Ensemble Model (SEM), a Transformer model, and a Residual Multi-layer Perceptron model. Additionally, the performance was compared with conventional 16S GCN correction algorithms. Once an optimal model was selected, the train datasets and test datasets were combined to train the final model.

A final test was conducted to evaluate the final model based on an independent test dataset from the newly released rrnDB 5.8. During this study, a new version of the database rrnDB 5.8 were released on June 23, 2022. This update added 8059 new 16S DNA sequences and their 16S GCN to the rrnDB database, including 219 genera that were not present in the previous rrnDB version 5.7. The newly added data were used as an independent dataset for the final test.

### Method comparison in cross-validation: conventional correction algorithms and existing tools

Currently, there are two types of conventional correction algorithms: taxonomy-based and phylogeny-based. Several conventional algorithms were implemented for cross-validation comparison. These algorithms were regenerated with the same train/test data as the machine learning models.

Taxonomic correction algorithms calculate the 16S GCN of a taxon from the mean of its sub-taxa^5,33^. To implement, 16S sequences were first classified using a naïve Bayesian classifier^34^ implemented in the *assignTaxonomy* function in the DADA2 package in R (version 1.21.0)^35^. Two well-established 16S databases, RDP (version 18)^36^ and Greengenes (version 13.8)^37^ were used as the reference database for the *assignTaxonomy* function to determine whether the choice of classification database would impact 16S GCN prediction performance. For method comparison, a taxonomic algorithm was implemented in Python (version 3.8) with an object-oriented programming approach. The algorithm took the taxonomic and 16S GCN data and treated every taxon as a Node object. Every Node is linked with its downstream Nodes (taxa), so eventually the Nodes form a hierarchical tree where the *Prokaryote domain* is the root, and the species are the tips. Finally, the algorithm pre-computed the 16S GCN value of every Node by taking the mean of its sub-Nodes. When a query is given, the algorithm will search the hierarchical tree for the lowest taxon of the query lineage. If a query has an unassigned taxon, it assigns the value of the query’s parent node to the query node as an estimation.

Phylogenetic algorithms were based on a pre-constructed tree of 16S rRNA gene sequences. The 16S GCN values of each ancestral node were estimated from its daughter species through weighing each labeled daughter node with phylogenetic distance to the parental node^38^. For method comparison, this study employed five phylogeny-based algorithms, including empirical probabilities (EP), subtree averaging (SA), phylogenetic independent contrasts (PIC)^39^, Sankoff’s maximum-parsimony (MPR)^40^, and weighted-squared-change parsimony (WSCP)^41^. To implement these algorithms, 16S rRNA gene sequences in the dataset were first aligned by MAFFT v7.490^42^ and then a 16S phylogenetic tree was constructed by FastTree 2.1.10^43^. The five phylogeny-based correction algorithms were then executed using the castor package in R^38^, with modifications made from the R script in^11^. In particular, MPR was implemented by the function *hsp_max_parsimony*, WSCP by the function *hsp_squared_change_parsimony*, SA by the function *hsp_subtree_averaging*, EP by the function *hsp_empirical_probabilities*, and PIC by the function *hsp_independent_contrasts*, with all parameters configured to default.

### Method comparison in model final test: existing correction tools

During the final test for ANNA 16, the existing correction tools, including rrnDB^5^, CopyRighter^6^, PAPRICA^44^, and PICRUSt2^12^, were implemented for method comparison. rrnDB is a taxonomy-based tool using taxonomic aggregation, while the latter three are phylogeny-based tools. CopyRighter uses the PIC algorithm to pre-compute the copy number of all taxa in the Greengenes database^37^ and makes predictions by searching the lineages of the queries in its pre-computed 16S GCN table^6^. PAPRICA and PICRUSt2 first utilize EPA-ng^45^ to place the queries on their built-in phylogenetic trees and then use subtree-averaging (PAPRICA) or maximum parsimony (PICRUSt2) to predict the 16S GCN of the queries^12,44^.

Because rrnDB and CopyRighter output copy number-corrected microbial community composition rather than 16S GCN values directly, this study extracted their precomputed tables (rrnDB version 5.7) to generate GCN values, searching the precomputed tables for assigned lineages. The taxonomy of full-length 16S sequences and all subregion sequences were generated by the *assignTaxonomy* function in the DADA2 package through RDP (version 18) and Greengenes (version 13.8, formatted by DADA2)^37^ database. The assigned RDP lineages were searched in the rrnDB precomputed table, and Greengenes lineages were searched in the CopyRighter precomputed table.

### Development of ANNA16

#### DNA sequence K-merization

**Figure 1:**
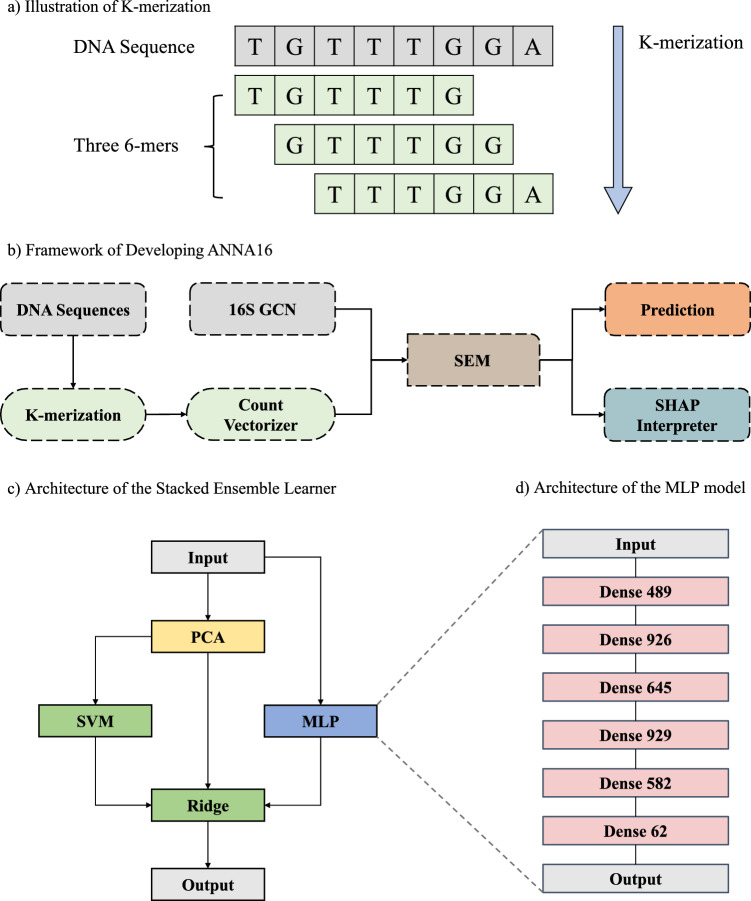
Illustration of the framework and architecture of the deep learning models. (**a**) DNA sequence K-merization (K = 6). (**b**) Framework for ANNA16. (**c**) Architecture of the MLP-based Stacked Ensemble Model (SEM). (**d**) Architecture of the MLP module inside the SEM.

The 16S rRNA gene sequences were first preprocessed with K-merization. An example of a set with three 6-mers is displayed in Fig. 1a. The K-mers were converted into the number of counts in the set via the *CountVectorizer* function in the scikit-learn module (version 0.23.2)^46^. Subsequently, the K-mer counts and 16S GCN data were used to train the SEM models.

After testing K from 3 to 7 on a prototype 3-layer Multi-layer Perceptron (MLP) model, the model performance was found to improve with increasing K. However, a larger K value required significantly more memory. Thus, the optimal K value was determined to be 6, striking a balance between good performance and manageable memory requirements.

#### Model construction

Based on 6-mers, a MLP based Stacked Ensemble Model (SEM) was constructed for 16S GCN prediction (Fig. 1b), using the Tensorflow package (version 2.8.0) in Python (version 3.8) on a Nvidia RTX 3090 GPU, with the initial dataset split by 3:1 for training and testing. This SEM method involves training multiple base models, after which a meta-model is used to aggregate the outputs of all base-models and make the final prediction. Typically, the stacked model performs better than any single base model^47,48^.

The first base model of SEM is a Multi-layer Perceptron (MLP). MLP is a type of artificial neural network that consists of layers of artificial neurons, and the input is processed layer by layer^16^. The calculation of the output for each neuron is as follows^16^:

$$\begin{aligned} \nu_{k} & = \mathop \sum \limits_{i = 0}^{n} \omega_{k,i} x_{i} \\ \gamma_{k} & = \varphi \left( {\nu_{k} + \nu_{{k_{0} }} } \right) \\ \end{aligned}$$

where *ν*_*k*_ is the net output of neuron *k*, with *x*_1_, *x*_2_, …, *x*_*n*_ being the input from the last layer, and $\omega_{k,1} ,\omega_{k,2} , \ldots ,\omega_{k,n}$ being the weights on the input. *γ*_*k*_ is the output of the neuron, where $\nu_{{k_{0} }}$ represents the bias, and *φ*(·)refers to the activation function. And the MLP part is trained with Rooted Mean Squared Error (RMSE) loss.

In addition to MLP, several classic machine learning algorithms were also tested as base-models, including Support Vector Machine (SVM)^49^, Random Forest^50^, K-nearest Neighbors^51^, and Gradient Boosting^52^. The K-mer counts data would first undergo a dimension reduction by the Principal Component Analysis (PCA) algorithm, implemented by the *PCA* function in scikit-learn with *n_components* set to 100, and then input to the machine learning models. Linear Regression, Ridge Regression^53^, and Lasso Regression^54^ were tested as meta-models which accept the output of MLP, PCA, and machine learning models and make the final prediction. All machine learning algorithms were implemented by the scikit-learn module (version 0.23.2). After the initial testing, SVM was selected among the base-models and Ridge Regression among the meta-models (Fig. 1c).

#### Hyperparameter optimization

The hyperparameters of SEM was optimized through the *SMAC4BB* function in the SMAC3 package (version 1.4.0)^55^ for 500 iterations. For the MLP component, hyperparameters including the number of dense layers, hidden layer size, activation functions, and learning rate were tuned. The number of dense layers of the model was tested from 1 to 7, hidden layer size from the range of [1, 1024], activation functions are selected among Rectified Linear Unit (RELU), Exponential Linear Unit (ELU), Gaussian Error Linear Unit (GELU), Scaled Exponential Linear Unit (SELU), and Linear, and the learning rate was tested in the range of [0.00005, 0.1]. The mathematic formulae of the activation functions are demonstrated in the Supplementary Methods.

The final MLP architecture (Fig. 1d) contains a dense block of 6 hidden layers, with neuron numbers of 489, 926, 645, 929, 582, and 82 respectively, and the activation functions are GELU, ReLU, ReLU, ELU, GELU, and Linear respectively. For SVM, the *kernel* value was chosen from “rbf”, “sigmoid”, and “linear”, *gamma* was tested between “scale” and “auto”, and *C* was tested as a float in the range of [0.3, 0.9] or as an integer in the range of [1, 100]. For Ridge, the value of *alpha* was tested as a float in the range of [0.3, 0.9] or as an integer in the range of [1, 100]. After tuning, the optimized parameters are *kernel* = *‘rbf’*, *gamma* = *‘auto’*, *C* = *11*, and *alpha* = *49* (a complete list of parameters is shown in Table S2).

The resulting final model is named the Artificial Neural Network Approximator for 16S rRNA gene copy number (ANNA16).

#### Other machine learning models tested

In addition to the SEM, this study also developed and evaluated a Transformer-based model, and a Residual Multi-layer Perceptron^56^, a ResNet34^57^, and a k-nearest neighbor model for 16S GCN prediction. Detailed methodologies and initial results for these models can be found in Supplementary Methods and Fig. S1. However, upon evaluation, neither model matches the performance of the MLP-based SEM model (Fig. S2).

### Mock community test

To assess model performance, an in silico test study was designed based on the known community structure of two commercially available mock communities^58^, one even community (ATCC, MSA-2003) and one staggered community (ATCC, MSA-1001) (Table S3). Both mock communities consist of ten bacterial strains in different ratios. This study retrieved the genome sequences of the ten strains from NCBI and copy number data from rrnDB (version 5.7). An arbitrary total cell number (1 × 10^5^ cells) was assigned to each community and the cell number for each strain was calculated based on the known/targeted fraction.

To evaluate the accuracy of community profile prediction by different tools, the Bray–Curtis Dissimilarity^59^ was calculated between the true cell fraction and the estimated cell fraction using the *vegdist* function in the vegan package (version 2.5-7).

### Model explanation

In this study, ANNA16 integrates three feature extractors: PCA, SVM, and MLP. The contribution of each feature extractor is first assessed by weighing the output of each feature extractor with the corresponding Ridge Regression coefficients, and then divided by the final ANNA16 output. This approach quantifies the percentage that each feature extractor contributes to the final output. In addition, SHAP, a unified measure of feature importance that quantifies individual feature contributions to a model prediction, was utilized to explain DNA loci’s contributions^28^. Based on collational game theory^60^ and the classical Shapley method^61^, SHAP views each feature as a player. The final contribution of a feature is the sum of all the marginal contributions across various subsets. Positive and negative SHAP values indicate positive and negative contributions to the model’s prediction, respectively. In this study, each K-mer is considered a feature, and its SHAP value is calculated using the *KernelExplainer* function in the shap module (version 0.41.0) in Python, treating every single prediction as an independent game.

For SHAP value analysis, 10% of the whole dataset was randomly sampled (N = 2758) and a list of SHAP values was assigned to every K-mer to represent the K-mer’s overall contribution. These SHAP values were then mapped back to the 16S rRNA gene sequence according to the position of the corresponding K-mers to investigate if there is a positional pattern in contribution. Furthermore, the rate of nucleic substitution, insertion, and deletion of each position was calculated using aligned 16S rRNA gene sequences, with *E. coli* (accession number: GCF_002953035.1) as the reference.

To explore how ANNA16 weighs an insertion, the upper boundary of the SHAP values versus the insertion rate was fitted with the elu function. To define the upper boundary, the insertion rate was first clustered with the *KMeans* function in the scikit-learn package into 50 ranks. For each rank, the mean insertion rate and max SHAP values were calculated. Adam optimizer in the tensorflow package was used to fit the elu function with a learning rate of 0.01 and 2000 iterations.

## Results

**Figure 2:**
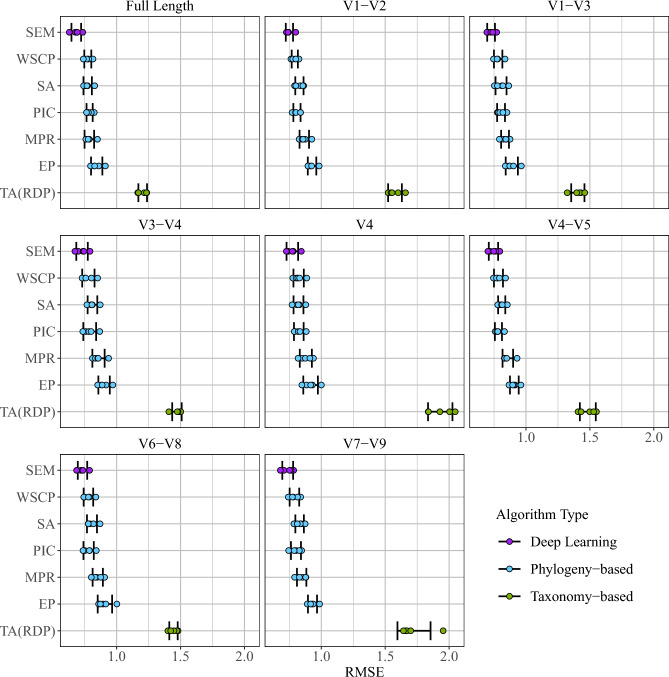
Comparison of prediction RMSE at 16S full-length and subregions. *EP* empirical probabilities, *MPR* Sankoff’s maximum-parsimony, *PIC* phylogenetic independent contrast, *SA* Subtree Averaging, *WSCP* weighted-squared-change parsimony, *TA(RDP)* taxonomic aggregation based on RDP database, *SEM* Stacked Ensemble Model.

For full length 16S rRNA gene sequences, the performance of the deep learning model SEM was evaluated in comparison with other conventional correction algorithms using fivefold cross validation (Fig. 2; Table 1). Among all compared algorithms, taxonomic aggregation methods (TA) perform the worst. TA based on RDP or Greengenes reference database exhibits a mean RMSE in copies/genome 1.18 (SD = 0.0356) and 2.52 (SD = 0.0388), respectively. Due to the much higher mean RMSE of TA with Greengenes, only results from TA with RDP are shown in Fig. 2. All phylogeny-based algorithms perform better than taxonomic aggregation, with WSCP the best phylogeny-based algorithm. SEM outperforms all other algorithms with the lowest mean RMSE of 0.685 copies/genome (SD = 0.0379), with an error range significantly lower than all other algorithms (p < 0.01).

**Table 1:** Summary of prediction RMSE at 16S full-length and subregions (mean RMSE ± SD, *p* value).

	Full length	V1–V2	V1–V3	V3–V4	V4	V4–V5	V6–V8	V7–V9
SEM	0.685 ± 0.0379	0.751 ± 0.0285	0.727 ± 0.0301	0.730 ± 0.0450	0.774 ± 0.0453	0.746 ± 0.0364	0.733 ± 0.0368	0.737 ± 0.0423
EP	0.845 ± 0.0443	0.928 ± 0.0331	0.889 ± 0.0476	0.902 ± 0.0447	0.916 ± 0.0569	0.910 ± 0.0340	0.909 ± 0.0563	0.931 ± 0.0354
	*p* < 0.001	*p* < 0.001	*p* < 0.001	*p* < 0.001	*p* < 0.01	*p* < 0.001	*p* < 0.001	*p* < 0.001
MPR	0.787 ± 0.0375	0.867 ± 0.0370	0.836 ± 0.0305	0.858 ± 0.0477	0.879 ± 0.0475	0.858 ± 0.0413	0.852 ± 0.0400	0.846 ± 0.0370
	*p* < 0.01	*p* < 0.001	*p* < 0.001	*p* < 0.01	*p* < 0.01	*p* < 0.001	*p* < 0.001	*p* < 0.01
PIC	0.789 ± 0.0243	0.810 ± 0.0281	0.805 ± 0.0307	0.789 ± 0.0503	0.824 ± 0.0377	0.785 ± 0.0277	0.781 ± 0.0404	0.802 ± 0.0393
	*p* < 0.001	*p* < 0.01	*p* < 0.01	*p* < 0.05	*p* < 0.05	*p* < 0.05	*p* < 0.05	*p* < 0.05
SA	0.774 ± 0.0329	0.829 ± 0.0321	0.805 ± 0.0430	0.811 ± 0.0379	0.822 ± 0.0388	0.810 ± 0.0283	0.807 ± 0.0391	0.830 ± 0.0334
	*p* < 0.01	*p* < 0.01	*p* < 0.01	*p* < 0.01	*p* = 0.054	*p* < 0.01	*p* < 0.01	*p* < 0.01
WSCP	0.775 ± 0.0273	0.793 ± 0.0242	0.782 ± 0.0330	0.779 ± 0.0483	0.823 ± 0.0405	0.785 ± 0.0354	0.779 ± 0.0377	0.790 ± 0.0375
	*p* < 0.05	*p* < 0.05	*p* < 0.05	*p* = 0.067	*p* = 0.054	*p* = 0.062	*p* < 0.05	*p* < 0.05
TA(RDP)	1.18 ± 0.0356	1.58 ± 0.0538	1.41 ± 0.0517	1.47 ± 0.0370	1.93 ± 0.0954	1.48 ± 0.0622	1.44 ± 0.0329	1.73 ± 0.129
	*p* < 0.001	*p* < 0.001	*p* < 0.001	*p* < 0.001	*p* < 0.001	*p* < 0.001	*p* < 0.001	*p* < 0.001
TA(Greengenes)	2.52 ± 0.0388	2.08 ± 0.122	2.63 ± 0.147	1.85 ± 0.0939	2.12 ± 0.0590	2.00 ± 0.0961	2.19 ± 0.232	2.54 ± 0.434
	*p* < 0.001	*p* < 0.001	*p* < 0.001	*p* < 0.001	*p* < 0.001	*p* < 0.001	*p* < 0.001	*p* < 0.001

*EP* empirical probabilities, *MPR* Sankoff’s maximum-parsimony, *PIC* phylogenetic independent contrast, *SA* subtree averaging, *WSCP* weighted-squared-change parsimony, *TA(RDP)* taxonomic aggregation based on RDP database, *TA(Greengenes)* taxonomic aggregation based on Greengenes database, *SEM* Stacked Ensemble Model.

Furthermore, algorithm performance was evaluated and compared for the commonly amplified 16S subregions in metabarcoding studies^30^. Compared to the performance on full length, in general, all algorithms produce larger RMSE on subregions (Fig. 2). SEM displays relatively consistent low RMSE on all subregions, ranging from 0.731 (V1–V3) to 0.774 (V4). Notably, except for V3–V4, V4, and V4–V5 of WSCP and V4 of SA, the error range of SEM on subregions is significantly lower than all other algorithms in all subregions (p < 0.05). The results indicate that SEM outperforms EP, MPR, PIC, and TA with RDP or Greengenes, while partially outperforming SA, and WSCP.

**Figure 3:**
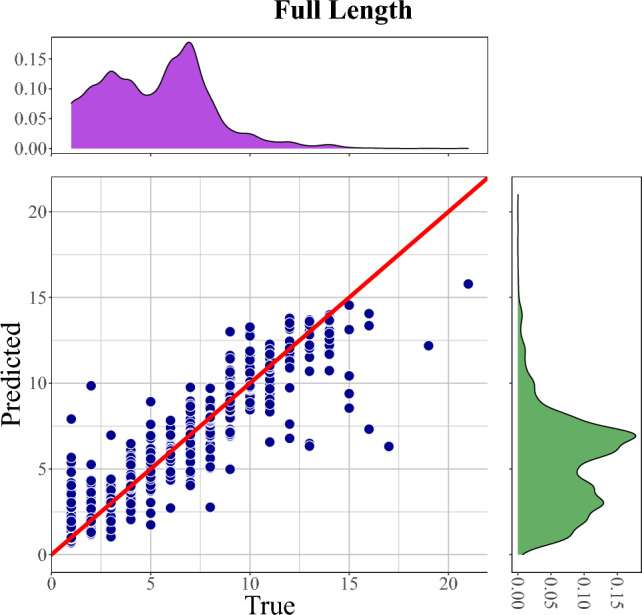
Prediction of 16S GCN using the MLP-based SEM on 16S full-length. Results from the first cycle of a fivefold cross-validation. The red 1:1 line illustrates deviation between predicted and actual values.

Overall, the 16S GCN prediction by SEM shows a strong linear relationship (Fig. 3, S3, and S6). However, SEM tends to underestimate the copy number when the true value is greater than 14. Due to SEM’s superior performance, after the cross-validation, the Artificial Neural Network Approximator for 16S rRNA gene copy number (ANNA16) is developed based on SEM using the full dataset.

**Figure 4:**
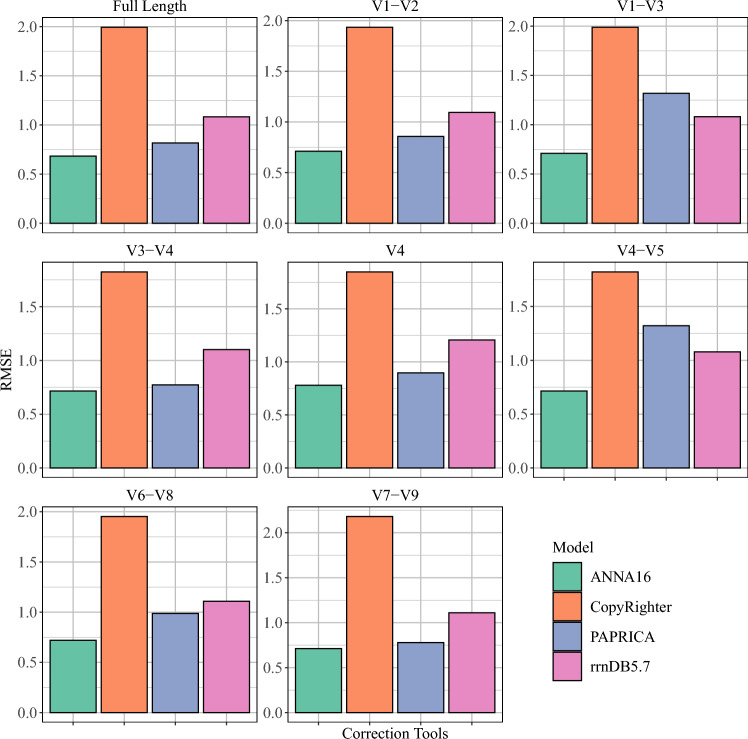
Summary of prediction RMSE in the final test. The prediction RMSE of ANNA16 was compared among the currently available correction tools, including CopyRighter, rrnDB5.7, PAPRICA, and PICRRUSt2 (details provided in Table 2). PICRUSt2 is mitted from this visualization due to its significantly elevated RMSE.

In the final test, ANNA16 was compared with the currently available correction tools (Fig. 4; Table 2). ANNA16 achieves the lowest RMSE among all the tools examined, with the smallest RMSE (0.683) at the full-length and the highest RMSE (0.780) at V4. In contrast, the RMSE of CopyRighter ranges from 1.822 (V4–V5) to 2.181 (V7–V9) copies/genome, rrnDB from 1.079 (V4–V5) to 1.207 (V4) copies/genome, PAPRICA from 0.773 (V3–V4) to 1.321 (V4–V5), and PICRUSt2 from 3.459 (V4–V5) to 4.731 (V6–V8). ANNA16 outperformed all other tools, demonstrating superior performance at all subregions and full-length, which indicated its superior ability to predict the 16S GCN values directly from DNA sequences.

**Table 2:** Performance of 16S GCN correction tools in the final test (RMSE).

	Full length	V1–V2	V1–V3	V3–V4	V4	V4–V5	V6–V8	V7–V9
CopyRighter	1.994	1.934	1.989	1.825	1.848	1.822	1.952	2.181
rrnDB5.7	1.083	1.094	1.082	1.101	1.207	1.079	1.109	1.110
ANNA16	0.683	0.711	0.710	0.716	0.780	0.715	0.720	0.713
PICRUSt2	4.198	4.353	4.175	4.393	3.807	3.459	4.731	4.677
PAPRICA	0.817	0.857	1.319	0.773	0.896	1.321	0.987	0.778

**Figure 5:**
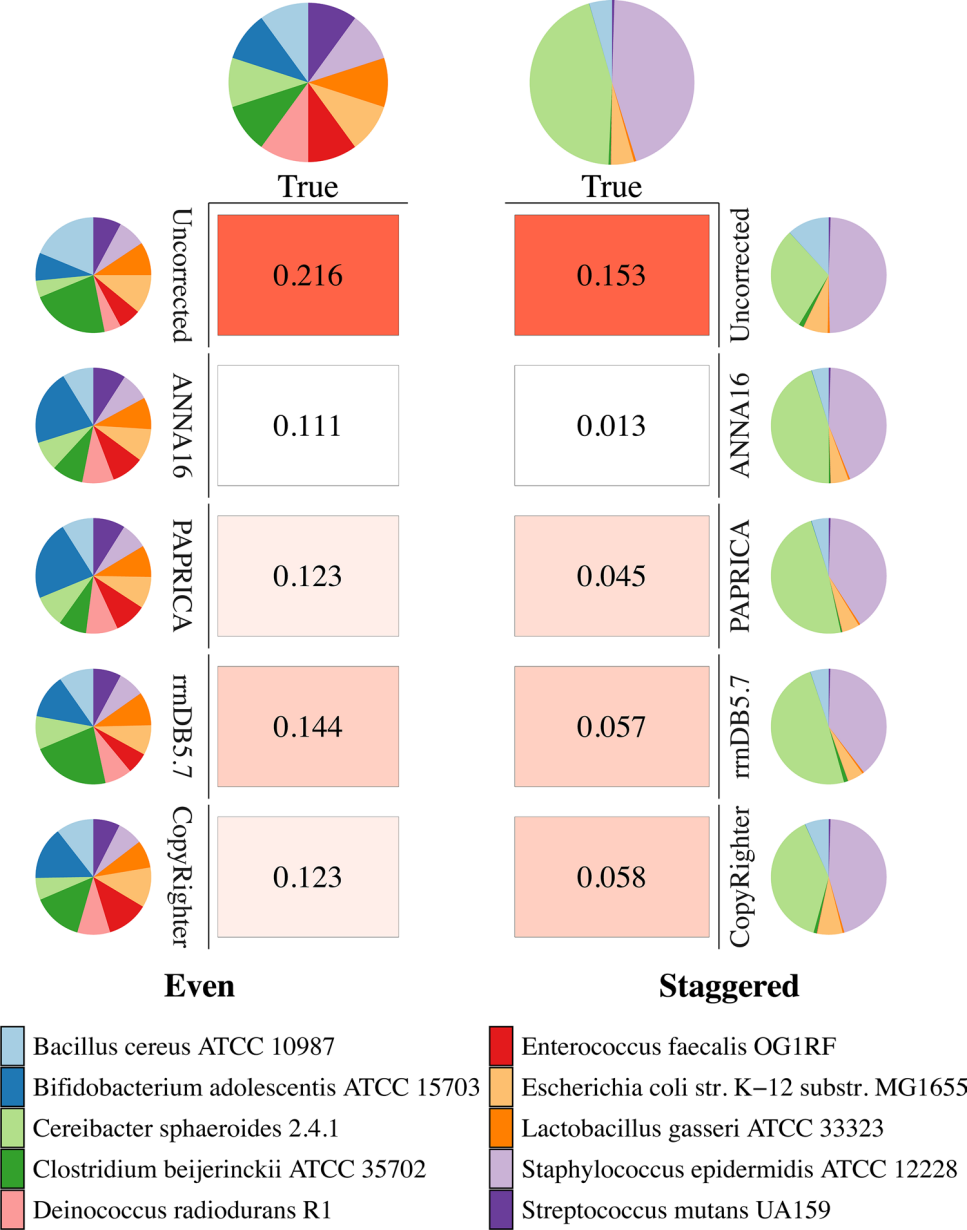
In silico test with the mock communities. The composition of the even community (left) and the staggered community (right) estimated by raw 16S sequencing reads, as well as copy number corrected reads estimated by ANNA16, PAPRICA, rrnDB (version 5.7), and CopyRighter. The Bray–Curtis Dissimilarity with the true cell composition is presented as the heatmap in the middle.

To demonstrate the application of ANNA16, an in silico case study of mock communities was conducted (Fig. 5; Tables S4 and S5). The significant difference between uncorrected 16S rRNA gene copy fractions and true cell fractions demonstrates that 16S GCN correction is necessary for quantitative microbiome profiling. In general, the Bray–Curtis dissimilarity with the true cell fractions for the even community is higher than those for the staggered community. The cell fractions estimated by ANNA16 have the smallest dissimilarity (Bray–Curtis) for both even and staggered communities.

**Figure 6:**
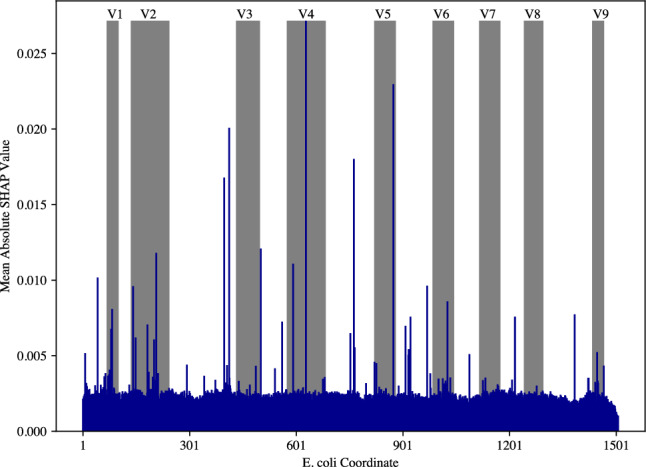
Mean absolute SHAP values across 16S rRNA gene. The mean absolute SHAP values, indicative of the DNA features contributing to the final model, were calculated for randomly sampled 10% of the aligned whole 16S rRNA full length dataset (N = 2758) and plotted on an *E. coli* 16S rRNA gene full length coordinate (accession number: GCF_002953035.1).

**Figure 7:**
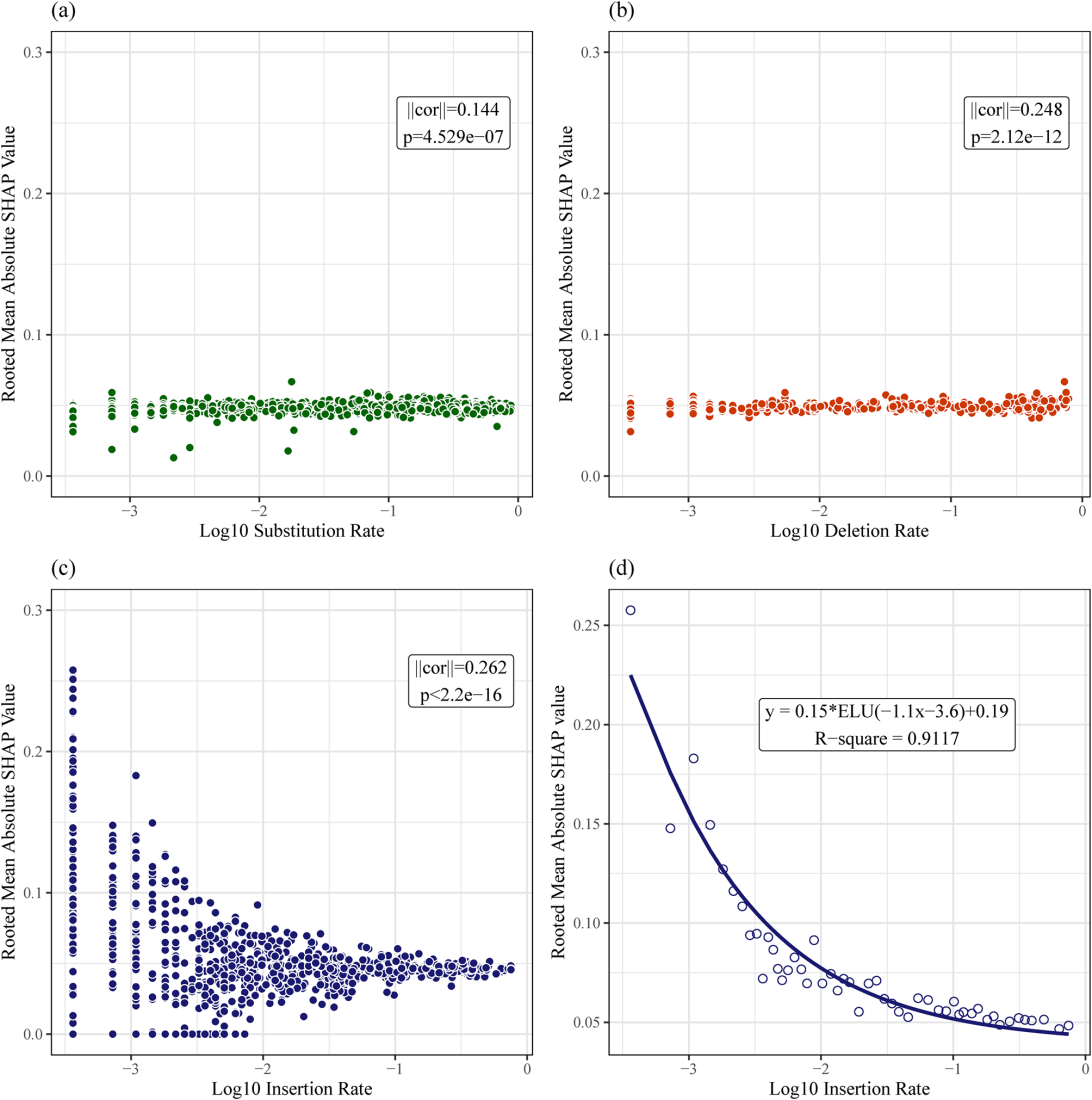
Relationship of SHAP values and mutation rates per locus. On each position of the aligned 16S rRNA gene sequences Rooted Mean Absolute SHAP values versus (**a**) Log10 substitution rates, (**b**) Log10 deletion rates, (**c**) Log10 insertion rates, and (**d**) the upper bound of Log10 insertion rates, fitted by an Exponential Linear Unit (ELU) function.

To decipher the mechanism underlying 16S rRNA gene copy number prediction based on DNA sequences, the contribution of each feature extract was first assessed. As shown in the Figure S5, MLP accounts for more than 75% of the output, with SVM playing a complementary role, while the contribution of PCA is small. In addition, Shapley Additive exPlanations (SHAP) — a game theory-informed method for model interpretation was applied to the full-length ANNA16 model. High absolute SHAR values correspond to positions that disproportionally contribute to the model’s final outcome. Interestingly, these positions do not align entirely with the conventionally identified 16S hypervariable regions (Fig. 6 and S4). No clear patterns observed between SHAP values and substitution or deletion rates (Fig. 7a,b). However, intriguingly, the insertion rates exhibit a non-linear relationship with SHAP values, with the upper bound of SHAP values decrease with increasing insertion rates (Fig. 7c). This trend aligns well with an exponential linear unit (ELU) activation function (Fig. 7d).

## Discussion

This study introduces ANNA16, a novel deep learning algorithm for predicting 16S GCN directly from on 16S rRNA gene sequences. This novel approach outperforms currently available taxonomy- and phylogeny-based algorithms in accuracy, excelling in predicting 16S GCN not only for the 16S full-length sequences but also in all commonly amplified subregions.

### Strengths of ANNA16

Compared with existing correction tools, the ANNA16 method owns their strengths and meanwhile avoids the drawbacks. Based on inference strategies, existing correction tools can be classified into pre-computation or real-time phylogenetic placement.

Pre-computational tools (i.e., rrnDB & CopyRighter) compute the 16S GCN value for every taxon in the database beforehand, and the tools retrieve the 16S GCN values from the database when a query is given. This strategy has the advantage of short inference time, but it is incompatible of differences in taxonomic databases nomenclatures. For example, the SILVA database^62^ merges *Allorhizobium*, *Neorhizobium*, *Pararhizobium*, and *Rhizobium* as one genus, named *Allorhizobium-Neorhizobium-Pararhizobium-Rhizobium*, but in RDP these are independent genera. If the classifier and 16S GCN predictor is trained with different nomenclatures, errors can be introduced.

On the other hand, real-time phylogenetic placement tools, such as PAPRICA and PICRUSt2, place a query sequence into their built-in phylogenetic trees to predict the 16S GCN values at runtime. This strategy yields prediction better than pre-computational tools, as shown in Figs. 2 and 4, but requires much longer inference time because phylogenetic placement can be computationally extensive. In contrast, ANNA16 not only outperforms these real-time phylogenetic placement tools but also maintains a short inference time, benefiting from the speed of GPU acceleration.

### ANNA16 as an explanatory deep-learning model for DNA

A fundamental question in bioinformatics concerns how models extract and interpret biological/evolutionary information contained within the biological sequences. The SHAP analysis suggest that the deep learning model ANNA16 may employ a unique approach.

The elevated SHAP values across the 16S full-length sequence do not correspond with the nine traditionally identified hypervariable regions (Fig. 6). While high absolute SHAP values can be observed within the V1, V2, V4, and V5 regions, the conserved regions preceding V1 and those between V2, V3, V4, V5, and V6 also contain loci with high absolute SHAP values, denoting their high informativeness to the model. In fact, some conserved regions appear to contribute more than certain hypervariable regions such as V3 and V6–V9. This indicates that for ANNA16, the conventional concept of base pair variability is not equivalent to informativeness.

Unexpectedly, SHAP do not exhibit any clear relationship with nucleic substitutions and deletions, two main parameters to infer evolutionary information in traditional phylogenetic methods^63,64^. Instead, an intriguing relationship emerges between SHAP values and the insertion rates. Elevated SHAP values aggregate at the positions with the lowest end of the insertion rates (Fig. 7c). This suggests that some infrequent insertions are the most informative for ANNA16.

Interestingly, the upper bound of SHAP values vs. the insertion rates can be fit with an ELU activation function (R^2^ = 0.91) (Fig. 7d), an activation function for neural networks and utilized in ANNA16. One potential explanation is that certain neurons in the MLP part of ANNA16 specialize in detecting these rare but informative insertions, and they are assigned high weights by other neurons. Like biological neurons, artificial neurons also receive input signals and fire output to other neurons if the stimulus reaches a certain threshold. Hypothetically, these specialized neurons may only fire when ANNA16 identifies an insertion whose rarity meets the threshold, approximately 10^−2^ per site (Fig. 7d).

In the aspect of information extraction, conventional taxonomic- or phylogeny-based correction algorithms could have two potential drawbacks. First, these algorithms typically weigh every position equally by converting the variance in the sequence-to-sequence similarity, which can underrepresent more informative positions. Second, some sequence information could be lost during the intermediate steps such as inferring a phylogenic tree. For instance, many tree-building methods ignore alignment gaps that contain biological information^64–68^. Previous studies have proved that indels are likely to experience few homoplasies and may produce more congruent phylogenetic trees if combined with substitutions^64–68^. ANNA16 adopts a more nuanced approach, which not only considers the substitutions but also pays special attention to insertions.

Beyond 16S GCN prediction, the efficacy of ANNA16 implies the broader potential of deep learning algorithms in functional biology studies. Deep learning models could provide precise trait predictions directly from biological sequence data. By evaluating the significance of each nucleotide or amino acid feature, these models can also be used to link specific loci to the traits of interest^69,70^.

### Limitations and future studies

One noted limitation of ANNA16 is its tendency to underestimate the species with high 16S GCN values. ANNA16’s predictions become increasingly skew lower when the actual 16S GCN exceeds 14 (Fig. 3 & S3). This disparity can also be observed in the predictions by other correction algorithms where the residues are observed to be more positive for low-copy number species, and more negative for high-copy number species (Fig. S6). This indicates the bias may arise from the skewed distribution of 16S GCN within the present dataset (Fig. S7). Sequences with more than 12 copies account for only a small proportion of the whole dataset, and sequences with more than 15 copies are extremely rare. Fortunately, as emerging sequencing methods and decreasing sequencing prices yield more genomic data, this limitation of ANNA16 could be resolved when the data of more species are available for training. Similar database bias challenges have been discussed by^6,11^. Alternatively, data imbalance methods, such as class re-balancing, information augmentation, and module improvement^71^ could be applied to future models. Taxonomic aggregation or pre-computational tools (Tables 1 and 2) may also enhance their performance through utilizing more sophisticated taxonomy classification algorithms^20,72,73^.

Furthermore, transfer learning^74,75^ presents another promising avenue. In the framework of transfer learning, a deep learning model is first pre-trained on one or more source tasks to capture general features or representation, and then is fine-tuned to apply the captured knowledge to target tasks^76^. To utilize this strategy, future studies could collect a large amount of unlabeled 16S rRNA sequences and pre-train a model with an autoregression or autoencoder task, similar to Large Language Models like BERT^77^ or GPT^78^, and then fine-tune the model on the child task, 16S GCN prediction, which may yield more accurate results.

## Conclusions

In conclusion, this study developed ANNA16 as a novel bioinformatic algorithm to predict the 16S rRNA gene copy numbers directly from DNA sequences. Compared to existing taxonomy-based and phylogeny-based algorithms, ANNA16 showed significantly lower error ranges at 16S full-length and all commonly amplified subregions. Furthermore, in the final test and mock community case study, ANNA16 outperformed existing correction tools such as rrnDB, CopyRighter, PICRUSt2, and PAPRICA. ANNA16’s structure offers several advantages, including faster inference speed and independence of pre-computation. SHAP analysis implies ANNA16 could utilize the information contained in the DNA sequences effectively by paying special attention to infrequent insertions.

### Supplementary Information

Supplementary Information.

## Data Availability

16S rRNA gene sequences and the gene copy number data used in the current study are available in the rrnDB repository, https://rrndb.umms.med.umich.edu/. The model weights of ANNA16 are available at https://github.com/Merlinaphist/ANNA16. ANNA16 is deployed on Google Colab, https://colab.research.google.com/drive/1XwpTMCHSfTmzpHyKrmiD8aC8C_1nndUV#scrollTo=M0jLLsHC9W4M. The codes for reproducing this study are available at https://github.com/Merlinaphist/ReproduceANNA16.

## Abbreviations

16S GCN: 16S rRNA gene copy number
ANNA16: Artificial Neural Network Approximator for 16S rRNA gene copy number
ELU: Exponential Linear Unit
EP: Empirical probabilities
GELU: Gaussian Error Linear Unit
MLP: Multi-layer perceptron
MPR: Sankoff’s maximum parsimony
PIC: Phylogenetic independent contrasts
RELU: Rectified Linear Unit
SA: Subtree averaging
SELU: Scaled Exponential Linear Unit
SEM: Stacked Ensemble Model
SHAP: Shapley Additive exPlanation
TA: Taxonomic aggregation
WSCP: Weighted squared change parsimony
