# Supplementary material for: Deep learning for predicting 16S rRNA gene copy number
Source: Sci Rep. 2024 Jun 20;14:14282. doi: 10.1038/s41598-024-64658-5 (PMC11190246; doi:10.1038/s41598-024-64658-5)
Supplement: Supplementary file 1 — Supplementary Information. [file 41598_2024_64658_MOESM1_ESM.pdf]

Supplementary Materials

Jiazheng Miao[1, 3], Tianlai Chen[1, 4], Mustafa Misir[1, *], and Yajuan Lin[1, 2, *]

[1]*Division of Applied and Natural Sciences, Duke Kunshan University, Suzhou, China*

[2]*Department of Life Sciences, Texas A&M University-Corpus Christi, Corpus Christi, USA*

[3]*Department of Biomedical Informatics, Harvard Medical School, Boston, USA*

[4]*Department of Biomedical Engineering, Duke University, Durham, USA*

*Correspondences:

Yajuan Lin

yajuan.lin@tamucc.edu

Mustafa Misir

mustafa.misir@dukekunshan.edu.cn

1 SUPPLEMENTARY METHODS

1.1.Mathematic Formulae of Activation Functions

$$RELU(x) = \begin{cases} 0, x \leq 0 \\ x, x > 0 \end{cases}$$

$$ELU(x, \alpha = 1) = \begin{cases} \alpha(e^x - 1), x \leq 0 \\ x, x > 0 \end{cases}$$

$$SELU(x, s = 1.05070098, \alpha = 1.67326324) = \begin{cases} s\alpha(e^x - 1), x \leq 0 \\ sx, x > 0 \end{cases}$$

$$GELU(x) = xP(X \leq x), where\ P(X) \sim N(0,1)$$

1.2.Other Machine Learning Models Tested

In addition to the SEM model, this study also tested a Transformer-based model and a Residual Multi-layer Perceptron (ResMLP), a ResNet34 [1], and a k-nearest neighbor model model. The framework and architecture of these models are shown in Figure S1.

1.2.1. Transformer

For the transformer-based model in Figure S1, we leveraged the original transformer encoder architecture with cosine learning rate decay [2], followed by global average pooling, and two dense layers. To help the transformer understand the genetic sequences, the K-mers were ranked by their frequency in the training data and the ranks were assigned as the indices. In this way, a sequence is converted into a list of tokens. As shown in Figure S1c, the transformer encoder takes embedding and position encoding as input. For biological sequences, a dense embedding is considered more effective than sparse tokenization. Each sequence is thus represented as a matrix $\mathbf{M} \in \mathbb{R}^{n \times d}$, by embedding each 6-mer into a numerical vector of

dimension *d* via the embedding layer. The cosine positional encoding method is used to incorporate both absolute and relative positional information by constructing the matrix **P** from **M** [3], where each position has:

$$p(2k+1, i) = \sin\left(i/1000^{2k/d}\right)$$

$$p(2k+2, i) = \cos\left(i/1000^{2k/d}\right)$$

The encoder includes multi-head self-attention, position-wise feed-forward network, residual connection, and layer normalization [3]. With the combination of these modules, the transformer encoder can process contextual information parallelly with the attention mechanism. For one single attention head, each token-embedding e_i is taken as query q_i to compute *scaled dot attention score* with all token-embedding in the same input sequence, i.e., $[e_1, e_2, \ldots, e_L]$, as key *k* and value *v*. Formally, the output of one attention head is defined as follows:

$$\text{head}(\boldsymbol{q}_i, (\boldsymbol{k}_1, \boldsymbol{v}_1), \ldots, (\boldsymbol{k}_L, \boldsymbol{v}_L)) = \sum_{j=1}^{L} \alpha(\boldsymbol{q}_i, \boldsymbol{k}_j)\boldsymbol{v}_j$$

$$\alpha(\boldsymbol{q}_i, \boldsymbol{k}_j) = \text{softmax}\left(\frac{\boldsymbol{q}_i{}^T \boldsymbol{k}_j}{\sqrt{d}}\right)$$

Thus, for the whole input sequence S, the attention score from the multi-head mechanism is

$$\mathbf{M} = \text{Multihead(S)} = \text{Concat}(\text{head}_1, \text{head}_2, \ldots, \text{head}_n)\mathbf{W}$$

where all **W** parameters are learnable for linear transformation. By learning different linear projections independently, it captures the combination of different representation subspaces. The intermediate representation is passed into the feed-forward network for further non-linear transformation with ReLU activation. The Layer Normalization is used here to avoid gradient explosion and accelerate convergence, and the Dropout method is to prevent over-fitting.

1.2.2. Residual Multi-layer Perceptron

ResMLP are MLP models utilizing Residual Connection architectures. In this architecture, skip connections are built to jump over two to three layers. Such design may help avoid the problem of vanishing gradients or degradation [4]. The ResMLP model is published in 2021 for image classification. It attains good accuracy without the presence of attention layer, bringing a surprise to the data science community [5].

The ResMLP model in this study is trained with Rooted Mean Squared Error (RMSE) loss:

$$RMSE = \sqrt{\frac{\sum_{i=0}^{n}(Y_{true} - Y_{pred})^2}{n}}$$

where n is the size of the input dataset, Y_{true} is the true value of copy number, Y_{pred} is the value of the predicted copy number.

Hyper-parameter tuning is performed on the number of dense blocks, the number of dense layers in a dense block, dense layer size, and learning rate. Hidden layer size is selected from the list of (128, 256, 512, 1024). The learning rate is tested from 0.001 to 0.002, with 0.0002 being a step. The final architecture of ResMLP for full-length DNA sequence is shown in Figure S1d & S1e.

1.2.3. k-Nearest Neighbor

Because each 16S sequence is represented as the count of K-mers, the k-nearest neighbor (kNN) model is added as a simple benchmark. This algorithm assigns the copy number of the closest training sequences as the copy number of a query. We implemented this algorithm by

the function *KNeighborsRegressor* from the sklearn package in Python. The copy number of the neighbors were weighed by the Euclidean Distance. The value of k was tuned from 1 to 7, and a final value of 5 is selected.

1.2.4. ResNet34

ResNet34 is a 34-layer convolutional neural network, with the residual connection bridges. This architecture is originally designed for image recognition [1], but is also found useful for protein annotation [6]. Onehot encoding is applied to the 16S sequences, so that they can be treated as an "image" and input into ResNet34. The details of the architecture implemented in this study is shown in Figure S1.

1.2.5. Model Performance

The performance of the machine learning models is shown in Figure S2. The ResNet34 model exhibits a mean RMSE of 0.965 copies/genome (SD = 0.132), Transformer equals to 0.861 copies/genome (SD = 0.0643), kNN equals to 0.764 copies/genome (SD = 0.0440), and ResMLP equals to 0.726 copies/genome (SD = 0.0381).

Notably, the performance of Transformer is significantly inferior to SEM ($p < 0.001$). This outcome is unexpected because Transformer-based models, such as BERT [7] or GPT [8], have achieved substantial success in the Natural Language Processing (NLP) field. A possible explanation might be related to the computing cost of the self-attention mechanism, which increases quadratically with sequence length, rendering Transformer-based models typically

unsuitable for modeling extended sequences [9]. For context, in NLP, classical models normally accept inputs below 512 or 1024 [7]. Since biological sequences are more complex than natural language and the input length surpasses one thousand, the subpar performance of the Transformer-based model is plausible. For future studies intending to utilize Transformer-based models to study 16S rRNA gene sequences, employing non-overlapping K-mer encoding, as was utilized in [10], might be beneficial in reducing the sequence length to a size manageable by the Transformer.

2 SUPPLEMENTARY TABLES

Table S1. Primers for Extraction of the 16S full-length and subregions [11, 12].

Regions	Forward Primers		Reverse Primers	
Full Length	27F	AGA GTT TGA TCC TGG CTC AG	1492R	TAC GGY TAC CTT GTT ACG ACT
V1-V2	27F	AGA GTT TGA TCC TGG CTC AG	338R	GCT GCC TCC CGT AGG AGT
V1-V3	27F	AGA GTT TGA TCC TGG CTC AG	534R	ATT ACC GCG GCT GCT GG
V3-V4	341F	CCT ACG GGA GGC AGC AG	785R	GAC TAC HVG GGT ATC TAA TCC
V4	515F	GTG CCA GCM GCC GCG GTA A	806R	GGA CTA CHV GGG TWT CTA AT
V4-V5	515F	GTG CCA GCM GCC GCG GTA A	926R	CCG YCA ATT YMT TTR AGT TT
V6-V8	939F	GAA TTG ACG GGG GCC CGC ACA AG	1378R	CGG TGT GTA CAA GGC CCG GGA ACG
V7-V9	1115F	CAA CGA GCG CAA CCC T	1492R	TAC GGY TAC CTT GTT ACG ACT

Table S2. Parameters of subunits in SEM.

**MLP Part**		
**Architecture**	**Number of Neurons (0, 1024)**	**Activation (ELU, GELU, SELU, ReLU)**
	489	GELU
	926	ReLU
	645	ReLU
	929	ELU
	582	GELU
	82	Linear
	1	Linear
**Learning Rate (5e-5, 1e-1)**	0.000167703	
**Batch Size (64, 128)**	100	
**Epoch (20, 60)**	59	
**Classic Machine Learning Part**		
**Model**	**Parameters**	**Value**
PCA	n_components	100
SVM	kernel (rbf, linear, sigmoid)	rbf
	gamma (auto, scale)	auto
	C (0.3-0.9, 1-100)	11
Ridge	alpha (0.3-0.9, 1-100)	49

The ranges tested during hyperparameter tuning were included in the parentheses.

Table S3. Information of mock communities.

Strain	GenBank ID	16S copy number	Cell Counts (Even)	Cell Counts (Staggered)
Bacillus cereus ATCC 10987	NC_003909.8	12	100,000	44,800
Bifidobacterium adolescentis ATCC 15703	NC_008618.1	5	100,000	400
Clostridium beijerinckii ATCC 35702	NC_009617.1	14	100,000	4,500
Deinococcus radiodurans R1	NC_001263.1	3	100,000	400
Enterococcus faecalis OG1RF	NC_017316.1	4	100,000	400
Escherichia coli str. K-12 substr. MG1655	NC_000913.3	7	100,000	44,800
Lactobacillus gasseri ATCC 33323	NC_008530.1	6	100,000	4,500
Cereibacter sphaeroides 2.4.1	NZ_AKVW01000001.1	3	100,000	44,800
Staphylococcus epidermidis ATCC 12228	NC_004461.1	5	100,000	44,800
Streptococcus mutans UA159	NC_004350.2	5	100,000	4,500

Table S4. Uncorrected, true, and estimated abundance of strains in even community.

Strain	Uncorrected	True	ANNA16	CopyRighter	rrnDB
Bacillus cereus ATCC 10987	0.1875	0.1000	0.0840	0.1055	0.0972
Bifidobacterium adolescentis ATCC 15703	0.0781	0.1000	0.2160	0.1473	0.1238
Clostridium beijerinckii ATCC 35702	0.2188	0.1000	0.0883	0.1408	0.2202
Deinococcus radiodurans R1	0.0469	0.1000	0.0923	0.0919	0.0763
Enterococcus faecalis OG1RF	0.0625	0.1000	0.0901	0.1171	0.0599
Escherichia coli str. K-12 substr. MG1655	0.1094	0.1000	0.0883	0.1124	0.0837
Lactobacillus gasseri ATCC 33323	0.0938	0.1000	0.0975	0.0776	0.0942
Cereibacter sphaeroides 2.4.1	0.0469	0.1000	0.0766	0.0616	0.0926
Staphylococcus epidermidis ATCC 12228	0.0781	0.1000	0.0760	0.0713	0.0739
Streptococcus mutans UA159	0.0781	0.1000	0.0909	0.0745	0.0781

Table S5. Uncorrected, true, and estimated abundance of strains in staggered community.

Strain	Uncorrected	TRUE	ANNA16	CopyRighter	rrnDB
Bacillus cereus ATCC 10987	0.1181	0.0448	0.0486	0.0668	0.0515
Bifidobacterium adolescentis ATCC 15703	0.0004	0.0004	0.0011	0.0008	0.0006
Clostridium beijerinckii ATCC 35702	0.0138	0.0045	0.0051	0.0090	0.0117
Deinococcus radiodurans R1	0.0003	0.0004	0.0005	0.0005	0.0004
Enterococcus faecalis OG1RF	0.0004	0.0004	0.0005	0.0007	0.0003
Escherichia coli str. K-12 substr. MG1655	0.0689	0.0448	0.0511	0.0712	0.0443
Lactobacillus gasseri ATCC 33323	0.0059	0.0045	0.0057	0.0049	0.0050
Cereibacter sphaeroides 2.4.1	0.2952	0.4479	0.4429	0.3899	0.4906
Staphylococcus epidermidis ATCC 12228	0.4920	0.4479	0.4393	0.4514	0.3915
Streptococcus mutans UA159	0.0049	0.0045	0.0053	0.0047	0.0042

3 SUPPLEMENTARY FIGURES

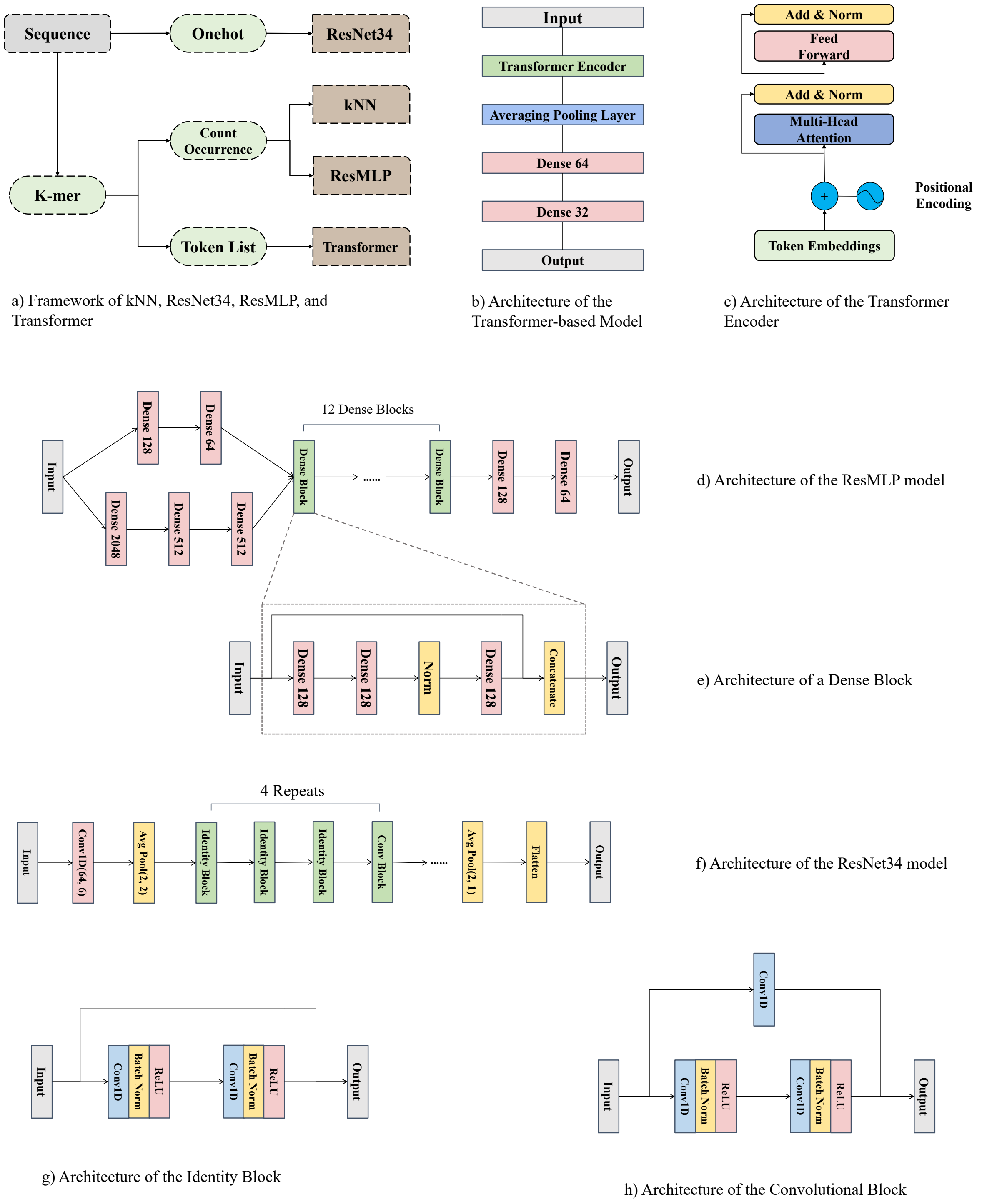

a) Framework of kNN, ResNet34, ResMLP, and Transformer

b) Architecture of the Transformer-based Model

c) Architecture of the Transformer Encoder

d) Architecture of the ResMLP model

e) Architecture of a Dense Block

f) Architecture of the ResNet34 model

g) Architecture of the Identity Block

h) Architecture of the Convolutional Block

Figure S1: Illustration of the Framework and Architecture of the Deep Learning Models.

a) Framework of kNN, ResNet34, ResMLP, and Transformer. b) Architecture of the Transformer-based model. c) Architecture of the Transformer Encoder. d) Architecture of the

ResMLP model. e) Architecture of a Dense Block inside the ResMLP model. f) Architecture of the ResNet34 model. g) Architecture of the Identity Block. h) Architecture of the Convolutional Block

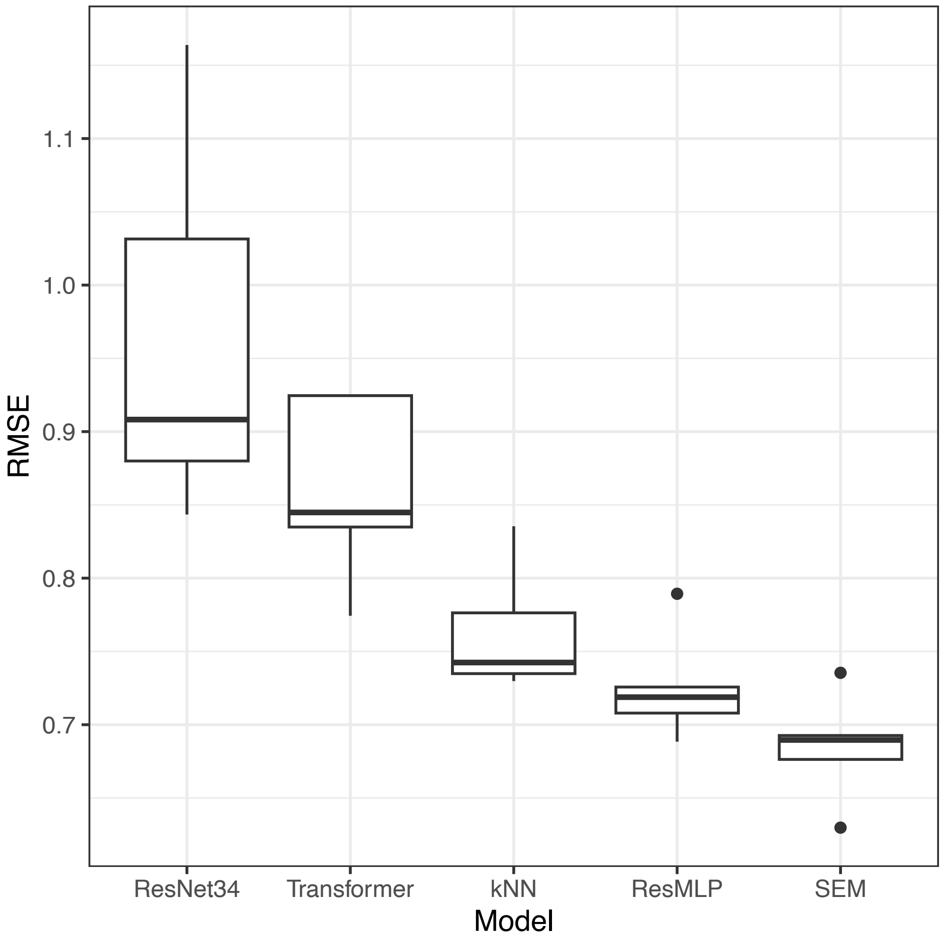

Figure S2: Performance of the Constructed Machine Learning Models.

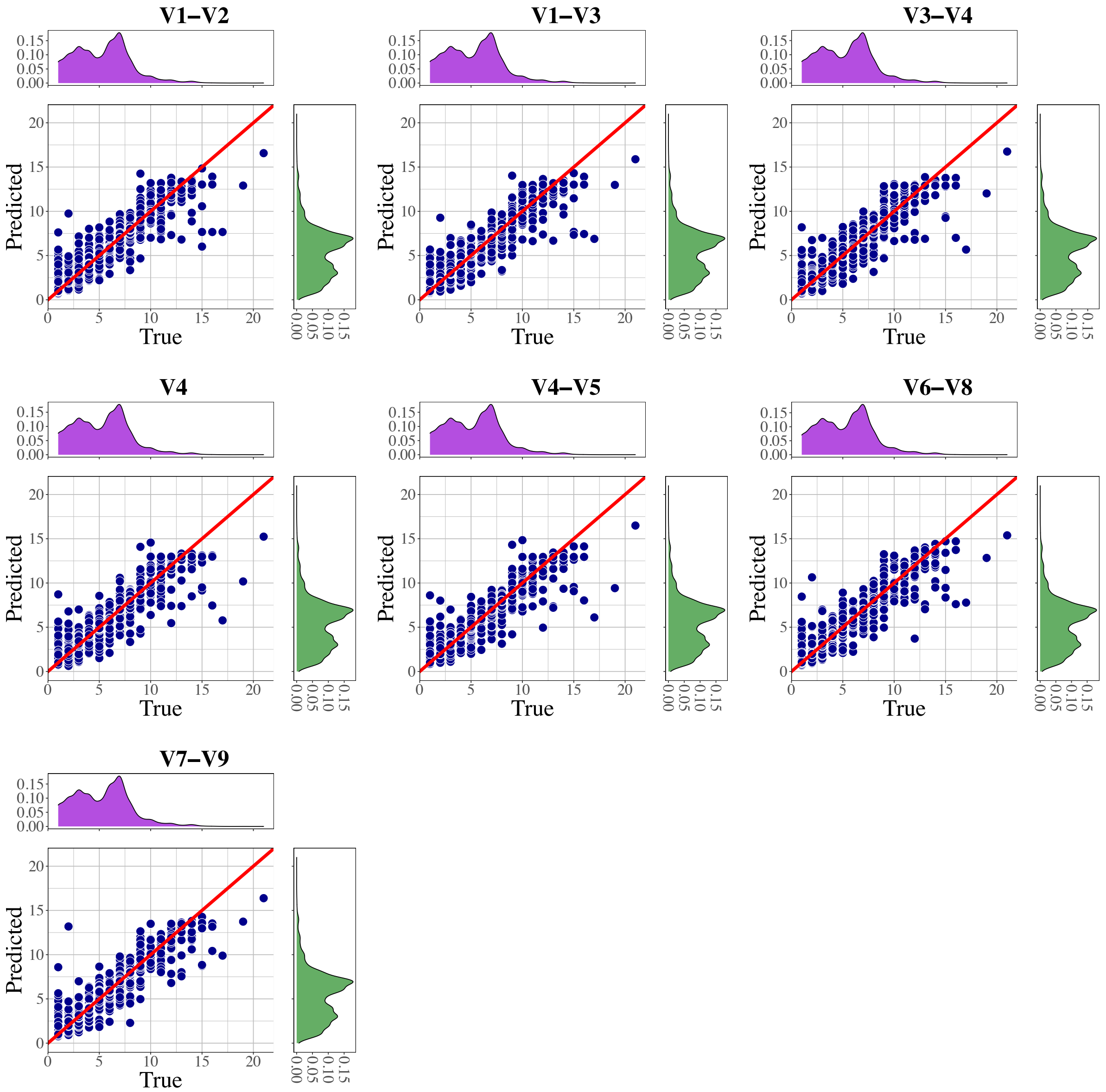

Figure S3. Prediction by SEM on 16S Subregions in the First Cycle of 5-fold Cross-validation. The red line is a diagonal line illustrating to which extent the predicted values are derived from the true values. The density plot filled in violet shows the distribution of the true values, and the green density plot shows the distribution of the predicted values.

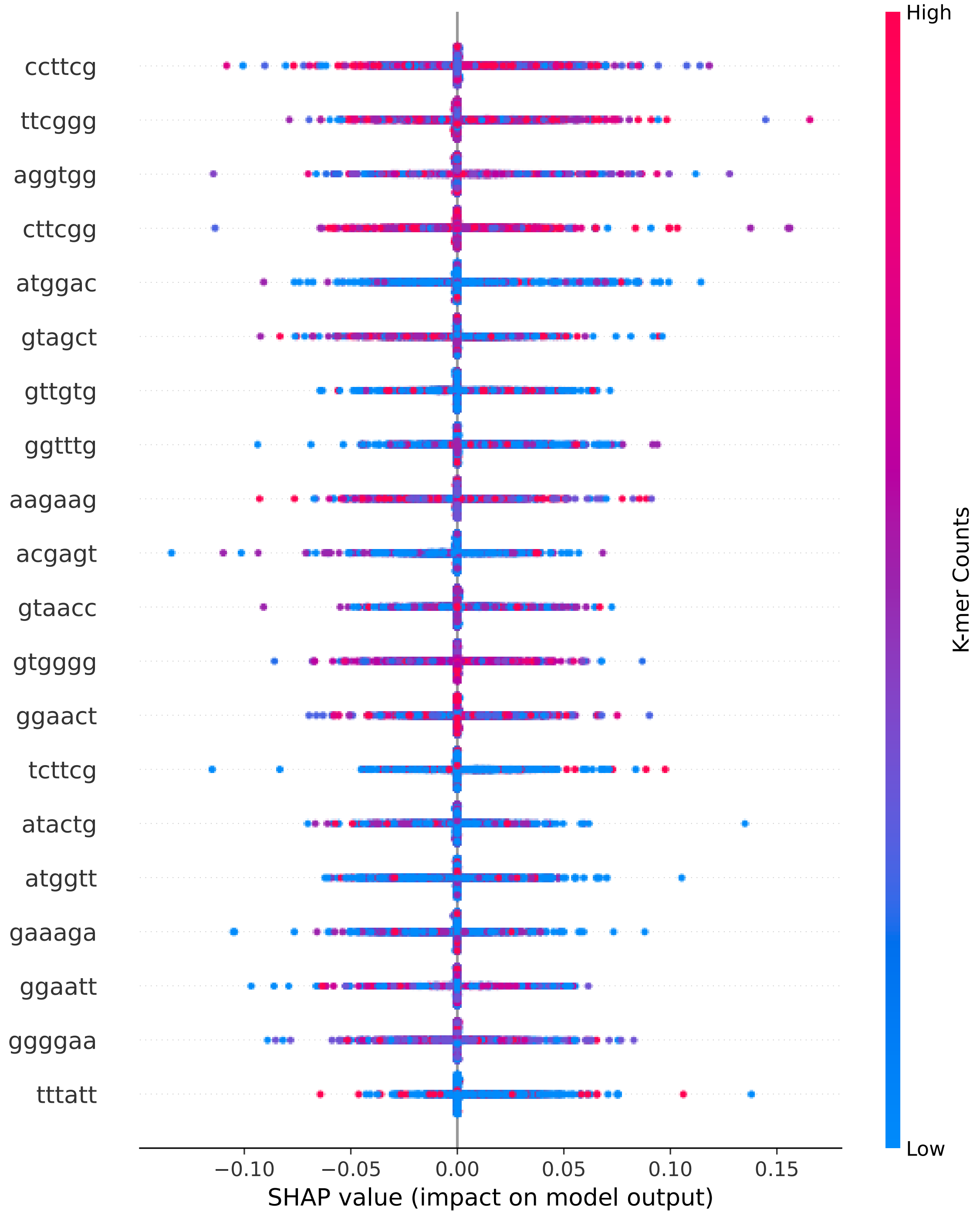

Figure S4. SHAP values of top 20 K-mers ranked by mean absolute SHAP value. Each point represents a SHAP value of a K-mer in a prediction.

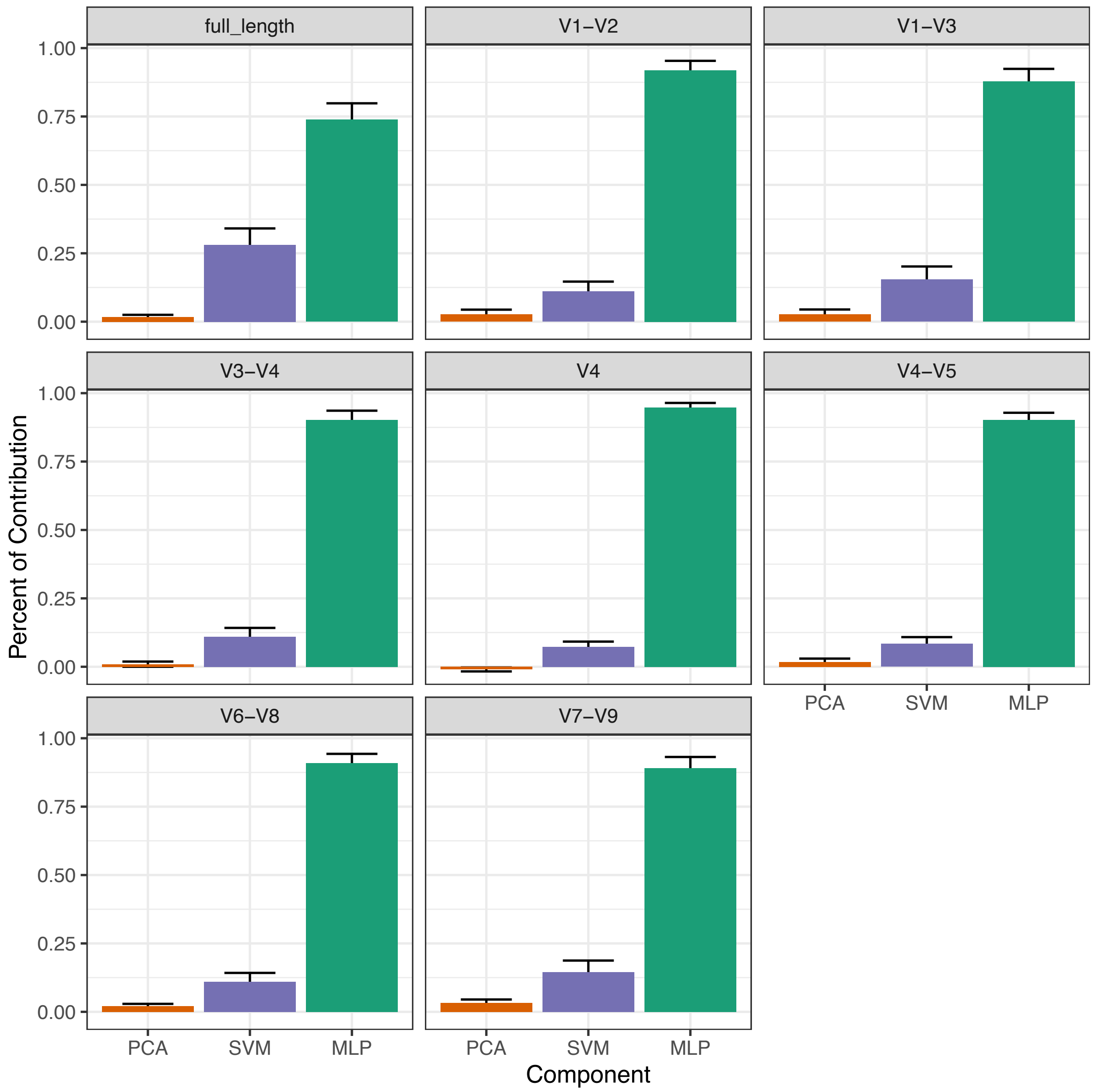

Figure S5. Contribution of each feature extractor in ANNA16.

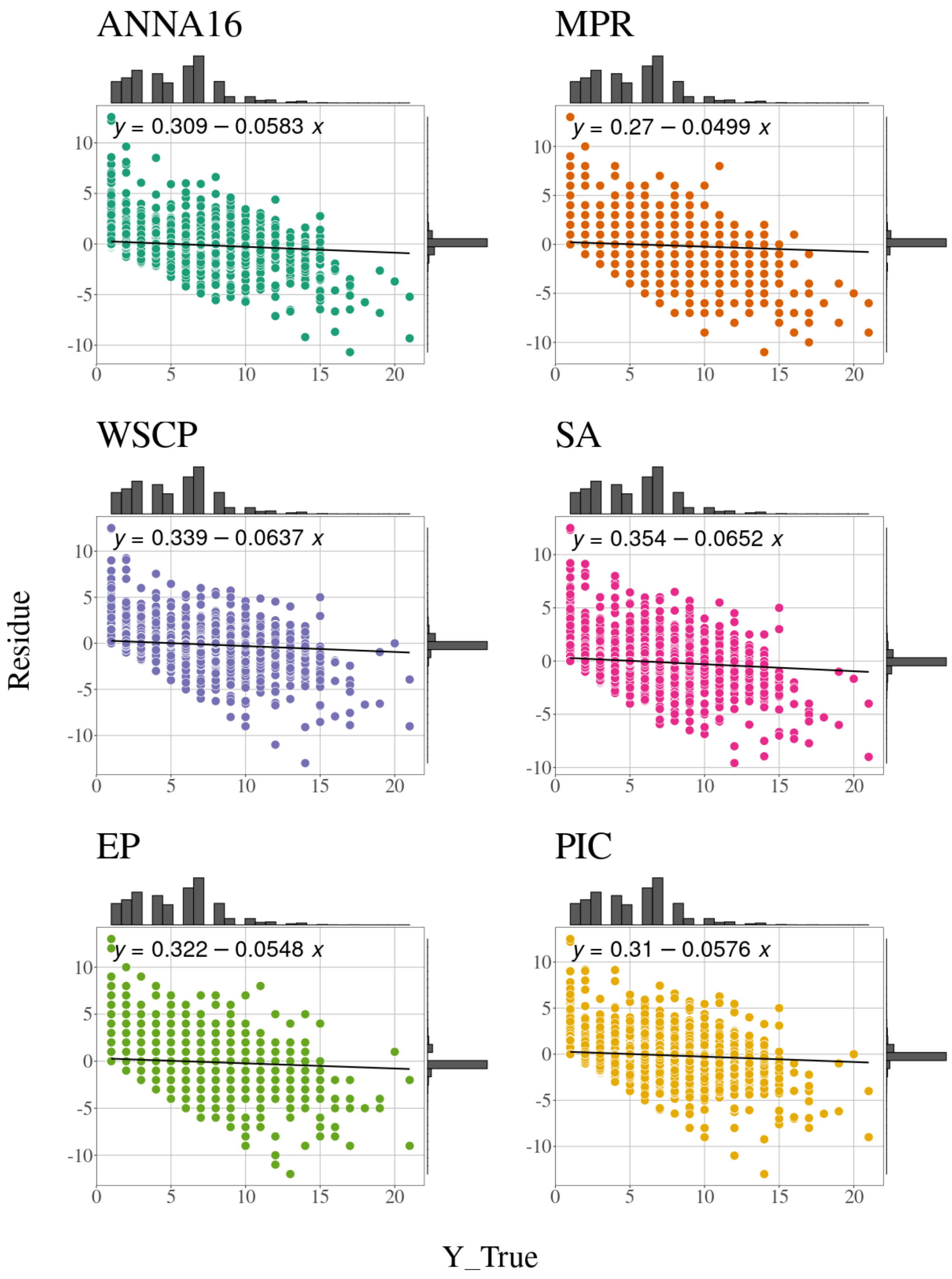

Figure S6. The relationship between the true copy number and prediction residues.

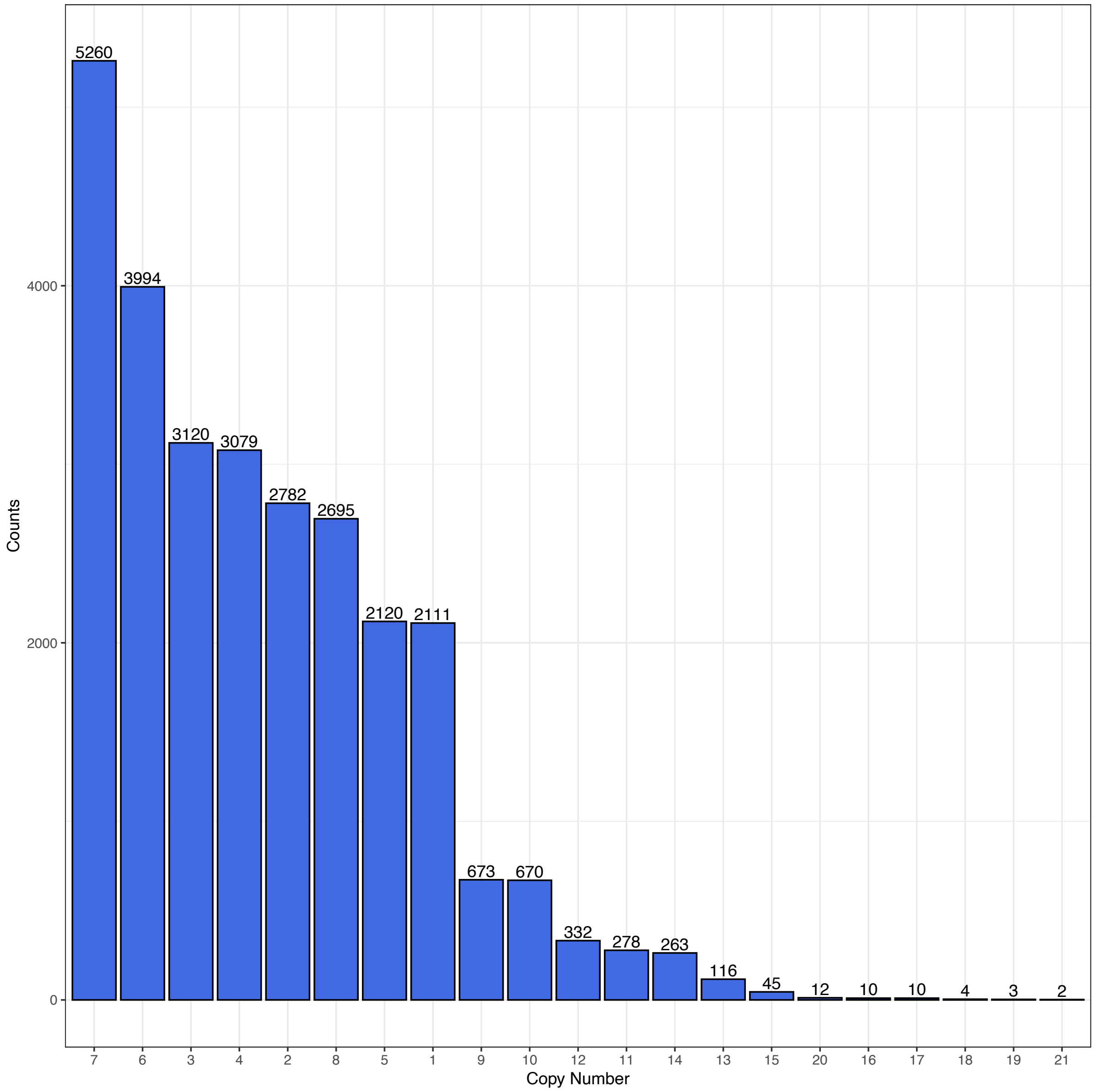

Figure S7. Distribution of the 16S copy number in the whole dataset.

REFERENCES

1. He K, Zhang X, Ren S, Sun J: **Deep Residual Learning for Image Recognition**. *arXiv [csCV]* 2015.
2. Loshchilov I, Hutter F: **SGDR: Stochastic Gradient Descent with Restarts**. *CoRR* 2016, **abs/1608.03983**.
3. Vaswani A, Shazeer N, Parmar N, Uszkoreit J, Jones L, Gomez AN, Kaiser L, Polosukhin I: **Attention Is All You Need**. *CoRR* 2017, **abs/1706.03762**.
4. He K, Zhang X, Ren S, Sun J: **Deep Residual Learning for Image Recognition**. In: *2016 IEEE Conference on Computer Vision and Pattern Recognition (CVPR): 27-30 June 2016 2016*. 770-778.
5. Touvron H, Bojanowski P, Caron M, Cord M, El-Nouby A, Grave E, Izacard G, Joulin A, Synnaeve G, Verbeek J *et al*: **ResMLP: Feedforward networks for image classification with data-efficient training**. 2021.
6. Bileschi ML, Belanger D, Bryant DH, Sanderson T, Carter B, Sculley D, Bateman A, DePristo MA, Colwell LJ: **Using deep learning to annotate the protein universe**. *Nature Biotechnology* 2022, **40**(6):932-937.
7. Devlin J, Chang M-W, Lee K, Toutanova K: **Bert: Pre-training of deep bidirectional transformers for language understanding**. *arXiv preprint arXiv:1810 04805* 2018.

8. Brown TB, Mann B, Ryder N, Subbiah M, Kaplan J, Dhariwal P, Neelakantan A, Shyam P, Sastry G, Askell A *et al*: **Language Models are Few-Shot Learners**. *CoRR* 2020, **abs/2005.14165**.

9. Beltagy I, Peters ME, Cohan A: **Longformer: The long-document transformer**. *arXiv preprint arXiv:2004 05150* 2020.

10. Mock F, Kretschmer F, Kriese A, Böcker S, Marz M: **Taxonomic classification of DNA sequences beyond sequence similarity using deep neural networks**. *Proc Natl Acad Sci U S A* 2022, **119**(35):e2122636119.

11. Abellan-Schneyder I, Matchado MS, Reitmeier S, Sommer A, Sewald Z, Baumbach J, List M, Neuhaus K: **Primer, Pipelines, Parameters: Issues in 16S rRNA Gene Sequencing**. *mSphere* 2021, **6**(1).

12. Parada AE, Needham DM, Fuhrman JA: **Every base matters: assessing small subunit rRNA primers for marine microbiomes with mock communities, time series and global field samples**. *Environmental Microbiology* 2016, **18**(5):1403-1414.
